# User fee exemption and malaria treatment-seeking for children under five in a Cameroonian health district: a cross-sectional study

**DOI:** 10.1186/s12936-023-04509-2

**Published:** 2023-04-13

**Authors:** Rolf Nyah Tuku Nzalie, John Ngunde Palle, Dickson Shey Nsagha

**Affiliations:** 1 Department of Public Health and Hygiene, Faculty of Health Sciences, University of Buea, Buea, Cameroon; 2 Department of Nursing, Faculty of Health Sciences, University of Buea, Buea, Cameroon

**Keywords:** Malaria, Treatment-seeking, Fee, Exemption, User, Guardians, Children

## Abstract

**Background:**

In Cameroon, malaria contributes significantly to the morbidity and mortality of children under 5 years old. In order to encourage adequate treatment-seeking in health facilities, user fee exemptions for malaria treatment have been instituted. However, many children are still brought to health facilities in the late stage of severe malaria. This study sought to determine the factors affecting the hospital treatment-seeking time of guardians of children under 5 years within the context of this user fee exemption.

**Methods:**

This was a cross-sectional study conducted at three randomly selected health facilities of the Buea Health District. A pre-tested questionnaire was used to collect data on the treatment-seeking behaviour and time of guardians, as well as potential predictors of this time. Hospital treatment sought after 24 h of noticing symptoms was denoted as delayed. Continuous variables were described using medians while categorical variables were described using percentages. A multivariate regression analysis was used to determine the factors affecting malaria treatment-seeking time of guardians. All statistical tests were done at a 95% confidence interval.

**Results:**

Most of the guardians made use of pre-hospital treatments, with self-medication being practiced by 39.7% (95% CI 35.1–44.3%) of them. A total of 193 (49.5%) guardians delayed seeking treatment at health facilities. Reasons for delay included financial constraints and watchful waiting at home, during which guardians waited and hoped their child could get better without requiring medicines. Guardians with estimated monthly household incomes denoted as low/middle were significantly more likely (AOR 3.794; 95% CI 2.125–6.774) to delay seeking hospital treatment. The occupation of guardians was another significant determinant of treatment-seeking time (AOR 0.042; 95% CI 0.003–0.607). Also, guardians with tertiary education were less likely (AOR 0.315; 95% CI 0.107–0.927) to delay seeking hospital treatment.

**Conclusions:**

This study suggests that despite user fee exemption, other factors such as educational and income levels of guardians affect malaria treatment-seeking time for children aged under five. Therefore, these factors should be considered when enacting policies aimed at increasing access of children to health facilities.

## Background

Despite recent reductions in its burden, malaria in sub-Saharan Africa (SSA) continues to be a major public health problem in children [1]. In 2018, children remained the most vulnerable group affected by malaria; those below 5 years of age accounted for 67% of all malarial deaths worldwide [1]. In Cameroon, although significant progress has been made to curb the impact of malaria, it is still the leading cause of morbidity and mortality in children [2]. Therefore, malaria contributes significantly to the high child mortality rate in Cameroon, situated at 80 per 1000 live births [3].

The clinical presentation of malaria in children usually ranges from the asymptomatic carriage of parasites, to a febrile disease that may evolve into a severe life-threatening illness [4]. Infected persons usually experience fever, headaches, joint pains, and malaise. Malaria infection has been classified into uncomplicated and severe malaria [5]. The latter can lead to a child’s demise, if treatment is delayed, due to the malaria parasite’s ability to induce complications such as cerebral malaria, severe anaemia and respiratory distress [6]. Other severe malaria manifestations that could be due to delayed treatment-seeking include prolonged seizures, circulatory collapse, hypoglycaemia, persistent vomiting, and intravascular haemolysis [7–9].

Delay in seeking treatment for malaria is described as when treatment is sought more than 24 h after recognition of symptoms [10]. To avoid complications from malaria, including the dreaded stage of severe malaria, it is important to adopt adequate treatment-seeking behaviour [11], and hence shorten the time taken to seek appropriate treatment [10]. One of the main components of the World Health Organization’s current strategy presses on the early recognition and prompt treatment of malaria [12]. Timely diagnosis, prompt and adequate treatment have been identified as the basis for the management of malaria and the key to reducing the burden of malaria, including its transmission [12].

Treatment-seeking time for children is determined by the treatment-seeking behaviour of their guardians, which is in turn determined by several social factors inherent to guardians [10, 11, 13]. Studies in Nigeria and India have identified factors such as the level of education, marital status, household income, age, and occupation of parents as being pivotal in determining the time taken to seek treatment [10, 14]. While these factors may promote or avoid delay in seeking treatment, the removal of user fees in health facilities has been noted to increase health services utilization [15], as well as reducing the time taken to seek malaria treatment [16, 17]. This is particularly true when the service seekers are cognizant of the existence of the user fee exemption policy [18].

In an effort to reduce the child mortality rate, the government of Cameroon has adopted several policies to reduce the burden of malaria—a major cause of child mortality in Cameroon [19]. One of such initiatives was the removal of user fees for the treatment of uncomplicated and severe malaria in children under 5 years of age, in all health facilities [19]. This initiative is in line with the fact that nowadays, user fee exemptions are seen as a means to improve access to health care for the most vulnerable population groups [15, 20, 21].

Since the implementation of the malaria user fee exemption policy in 2011, there has been an increase in malaria-related consultations for children aged 5 years and below [16, 17]. Despite this, some studies show that most children still arrive at health facilities in the late stage of severe malaria [16, 17]. Therefore, it is important to determine the factors that could be favouring delay in seeking malaria treatment for these children.

The aim of this study was to determine the factors affecting the seeking of hospital-based treatment by guardians of children under 5 years with malaria, in the context of a user fee exemption, in the Buea Health District.

## Methods

### Study area

**Fig. 1:**
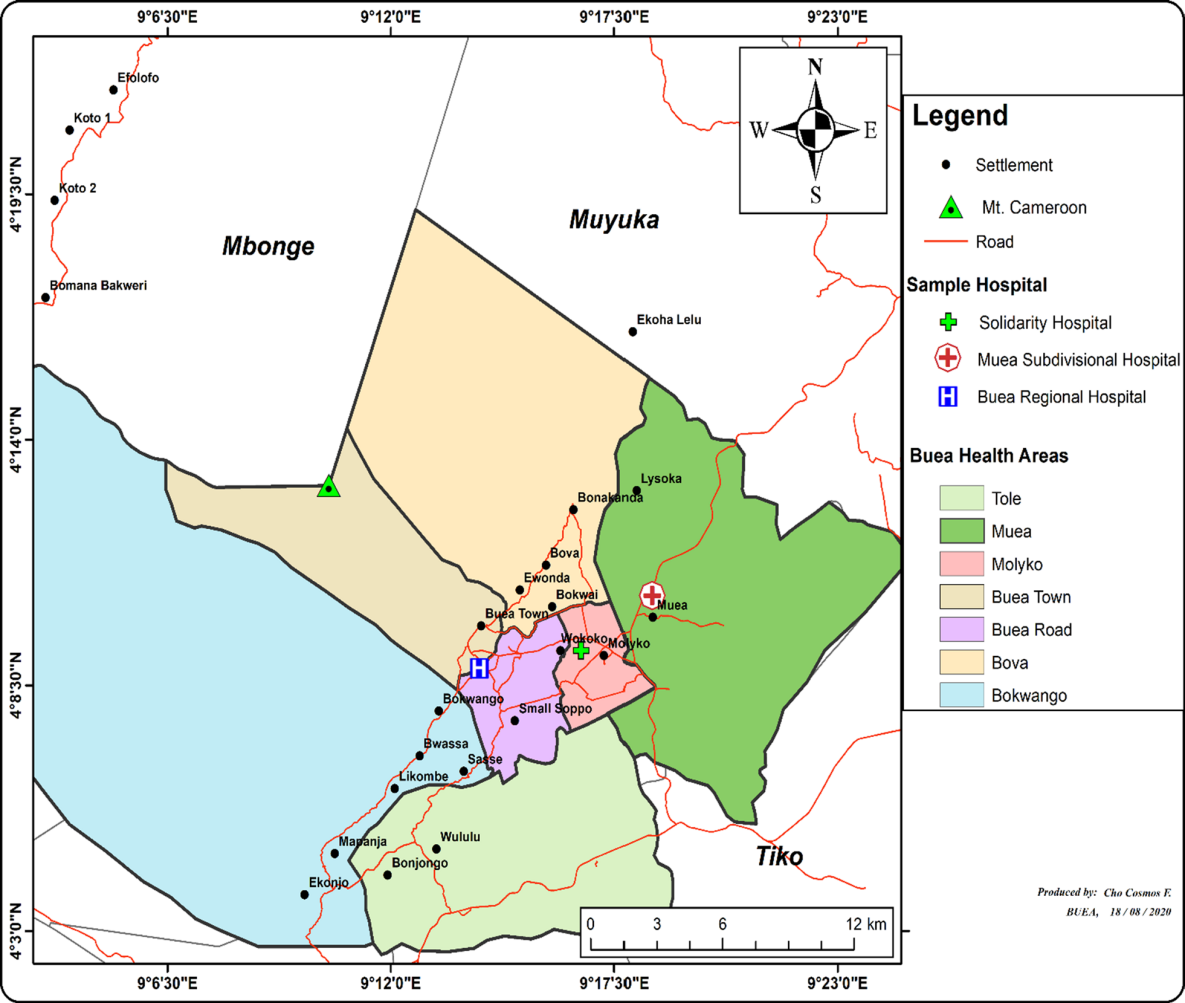
The Buea Health District (Source: principal investigator)

The Buea Health District (BHD) is located in the southwest region of Cameroon. This region has a total of 18 health districts, with that of Buea situated on the eastern slopes of Mount Cameroon, with a total surface area of 870 sq km. Buea has an equatorial climate with two seasons: the dry and wet seasons. Malaria transmission is most prominent during the wet season. In 2017, the BHD had an estimated population of 169,745 inhabitants; children under 5 years made up 13.6% of the population. The BHD is made up of seven health areas: Buea road, Muea, Molyko, Bova, Buea Town, Bokwaongo and Tole [22]. Figure 1 shows a map of the BHD.

### Study setting and duration

This study was carried out in three health facilities of BHD, selected through a stratified probabilistic sampling technique. The district has public, private and confessional health facilities, each having the capacity to diagnose and treat malaria in children. This study ran from January 2020 to July 2020, making a total of seven months.

### Study design and target population

This was a hospital-based, cross-sectional-study. The target population was the guardians of children under 5 years old with malaria, presenting at health facilities in BHD during the time of the study. Included were consenting adult guardians of children aged 5 years and under, with a diagnosis of malaria. Excluded were guardians of children who had malaria and another chronic or acute illness, guardians having more than one sick child at the hospital, and guardians with children referred from other health facilities.

### Sample size determination

With the prevalence of malaria in children estimated at 27.4% in the southwest region of Cameroon [5], the target population of guardians of children with malaria is assumed to be large. Hence, the sample size was calculated using Cochran’s formula for a large population [23].

Cochran formula:

$$n=\frac{{z}^{2 }p (1-p)}{{e}^{2}}$$

where, z: 1.96 at a 95% Confidence Interval; p: With proportion of guardians of children under 5 years with malaria in the general population not known, an estimate of 50% was considered; e: Desired precision (5%, i.e. 0.05); n: Minimum sample size.

Substituting the values, $n=\frac{{1.96}^{2 }0.5 (1-0.5)}{{0.05}^{2}}$n = 384 participants

### Sampling technique

A multistage sampling technique was used to select three health facilities in which the study was conducted. All the health facilities of the district were first divided into seven clusters, each representing a health area. Three health areas (Muea, Molyko and Buea road) were then selected by balloting. Subsequently, one health facility was selected by a second stage of balloting, from each of the selected clusters. The study was then conducted in these three selected health facilities, that is, the Muea Sub-divisional Hospital from the Muea health area, Solidarity Hospital from the Molyko health area, and the Buea Regional Hospital from the Buea road health area.

Table 1 shows the probability proportional to size used to determine the required number of participants per health area selected. This was done by dividing the number of children aged 5 years and below from each health area by the total from the three selected health areas. Based on these calculations, the proportion of participants per health area selected was 0.156 for Molyko health area, 0.462 from Muea health area, and 0.382 for Buea road health area.

**Table 1:** Probability proportional to size of health area

Health area (Health facility)	Total population ≤ 5 years	Proportion	Proportionate sample
Buea Road (Buea regional hospital)	6,588	0.382	149
Molyko (Solidarity hospital)	2,676	0.156	61
Muea (Muea Sub-divisional hospital)	7,993	0.462	180
Total	17,257	01	390

A consecutive sampling technique was employed to recruit participants from the three selected health facilities.

### Data collection tool

Data were collected with the help of a pre-tested structured questionnaire. Pre-testing was carried out on 27 participants from Molyko Health Centre, located in the study health district. During pre-testing, participants were asked to remark on their comprehension of the questions. After the process, some questions were rephrased in order to ease comprehension. This questionnaire (see Appendix 1) was divided into four parts.

The first part identified the participant with a code. It gathered information on the child’s age. The second part captured data on guardians’ socio-demographic characteristics such as age, gender, marital status, residence, and educational level, among others. The third part gathered data on socio-economic traits such as monthly income and occupation of guardians. The fourth part collected data on malaria treatment-seeking behaviour of guardians, and the time taken to seek hospital-based treatment, as well as reasons for delay. Early treatment-seeking referred to treatment sought within 24 h of the guardian noticing their child’s symptoms, while delayed treatment-seeking was considered as seeking treatment more than 24 h after noticing the symptoms [10, 12].

### Data collection

Trained data collectors proceeded daily to identify potential study participants and administer the questionnaire to them. The principal investigator (PI) carried out daily onsite supervision of data collection, and checked all answered questionnaires for consistency. Whenever inconsistencies were noted, the PI made sure the right information was obtained by interviewing the participant again. Data collectors received a financial motivation during training and as data collectors.

The pre-tested structured questionnaire was administered to consenting guardians of children under 5 years with malaria, who fulfilled the inclusion criteria. In a situation where more than one guardian existed, priority was given to the guardian present at the beginning of the child’s illness. The questionnaires were administered in English or Pidgin English, depending on the language the respondent best understood.

### Data management and statistical analysis of data

Questionnaires were checked daily for completeness and consistency by the PI. Code numbers were used to identify participants, and data were entered into Epi Info version 7.0 before being exported to Statistical Package for Social Sciences (SPSS) version 20.0 for analysis.

Continuous variables were first checked for normality using the Kolmogorov–Smirnov test, and normality transformations attempted, before being described using medians and interquartile ranges. Correlation techniques were used to evaluate relationships between continuous variables. Categorical variables were expressed using percentages, contingency tables, pie charts, and bar charts. The Pearson Chi-squared test was used to assess statistical significance of the categorical variables after making sure the smallest expected value exceeded five. In cases where this condition was not fulfilled, the likelihood ratio was considered. A multivariate logistic regression analysis was then carried out using treatment-seeking time (classified as delayed or early) as the outcome variable. The procedure for developing the multivariate model was as follows: a bivariate logistic analysis was first conducted for each of the independent variables, and then all those variables with *p-values* < 0.2 were considered candidates for the multivariate logistic model. All statistical tests were done at a 95% confidence interval, and *p-values* < 0.05 were considered statistically significant.

## Results

### Participants’ characteristics

A total of 402 guardians were recruited, of which 12 were excluded as their children were found to have other infections and chronic co-morbidities. A total of 390 guardians were finally included. Out of the 390 participants, 270 (69.2%) were female. The median age of the participants was 32 years, with an interquartile range of 7.3. As shown on Table 2, the most represented age group was the 20–35 years (67.2%), while the ≥ 68 years age group was the least represented. Most (62.3%) of the guardians mentioned they had a high estimated household monthly income (Table 2). With respect to marital status and educational level, most of the guardians (57.9%) were monogamously married, with a secondary/high school (46.7%) educational level (Table 2).

**Table 2:** Socio-demographic characteristics of guardians

Variables	Number of guardians	Percentage (%)
Marital status		
Widowed	17	4.4
Single	39	10.0
Polygamy	13	3.4
Monogamy	226	57.9
Divorced/separated	20	5.1
Co-habiting	75	19.2
Total	390	100.0
Level of education		
Primary	45	11.5
Secondary/High school	182	46.7
Tertiary	161	41.3
None	2	0.5
Total	390	100.0
Age groups (years)		
20–35	262	67.2
36–51	110	28.2
52–67	17	4.4
≥ 68	1	0.3
Total		
Occupation		
Government employed	61	15.6
Housewife	37	9.5
Private sector employed	112	28.7
Self-employed	149	38.2
Student	21	5.4
Unemployed	10	2.6
Total		
Estimated monthly household income [F CFA]		
High ( (≥ 101,000)	243	62.3
Middle (37,000–100,000)	146	37.4
Low (≤ 36,000)	1	0.3
Total	390	100

Table 2 shows the distribution of occupations among our participants. Participants were mostly self-employed (38.2%), with state employees representing 15.6%.

### Treatment-seeking behaviour of guardians of children with malaria

With respect to treatment-seeking behaviour, 242 guardians representing 62.1% (95% CI 57.0–66.9%; *p* = 0.000) of participants employed some other form of treatment before seeking hospital-based care. As shown on Table 3, stratifying usage of non-hospital care by age groups, the 20–35 years age group was least inclined (55.7%) to seek non-hospital-based treatment.

**Table 3:** Treatment-seeking behaviour of guardians

Non-hospital care	Age groups of guardians (years)				Likelihood ratio (14.657)
	20–35 No. (%)	36–51 No. (%)	52–67 No. (%)	≥ 68 No. (%)	
No	116 (44.3)	28 (25.5)	4 (23.5)	0 (0.0)	*p* = 0.002
Yes	146 (55.7)	82 (74.5)	13 (76.5)	1 (100.0)	
Total	262 (100)	110 (100)	17 (100)	1 (100)	

Reasons for delay	No.	Percentage (%)
Watch the child for a while	134	46.2
Financial contraints	136	46.9
Hospital is far	0	0.0
Use of alternative health care	16	5.5
Other reasons	4	1.4
Total	290	100.0

Sources of non-hospital based treatment	No.	Percentage (%)
Self-medication	96	31.8
Roadside vendor	45	14.9
Pharmacy	91	30.1
Herbalist	20	6.6
Family or friend	50	16.6
Other	0	0.0
Total	302	100.0

Table 3 illustrates the different sources of non-hospital-based treatment. Among guardians who sought treatment before arrival at a health facility, self-medication (39.7%) was the most common treatment-seeking behaviour, followed closely by visiting a pharmacy to obtain treatment (37.6%). In 18.6% of guardians who sought non-hospital care, a roadside drug vendor was consulted, while the services of a herbalist were requested by 8.3%.

### Reasons for delayed hospital arrival of children with malaria

A total of 193 [ (49.5%); 95% CI 44.4–54.6%; *p* = 0.041] guardians recorded a hospital treatment-seeking time over 24 h (i.e., delayed hospital arrival). Table 3 illustrates guardians’ reasons for delayed hospital arrival. Most delayed due to financial constraints (69.4%) and their desire to monitor their sick child (68.4%) in the hope of him/her getting better, especially when home remedies had been administered. No guardian mentioned distance to the health facility as a reason for delay in seeking treatment. In 2.0% of cases, other reasons for delay were cited as inability to seek hospital care due to insecurity, and the guardian being sick.

### Determinants of guardians’ treatment-seeking time for malaria in children

#### Socio-demographic characteristics and treatment-seeking time

Statistical relationships between the socio-demographic characteristics of guardians and the malaria treatment-seeking time for their children are depicted on Table 4. Most (73.6%) of the guardians who sought treatment within 24 h were female. Guardians aged 20–35 years represented the majority (77.2%) of those who sought treatment within the first 24 h of noticing symptoms, while those aged 36–51 years were the majority seeking treatment after 72 h. Also, a statistically significant relationship was noted between marital status and treatment-seeking time. Here, 53.3% of those who sought treatment within 24 h were monogamously married, whereas polygamous and single guardians represented only 1.5% and 14.7%, respectively. A statistically significant relationship was found between the level of education of guardians and treatment-seeking time. Most (59.4%) of those seeking care within 24 h had attained tertiary educational. The majority (61.4%) of those seeking treatment within 25–48 h were of secondary/high school level, while guardians with primary educational level represent 45% of those seeking treatment between 49 and 72 h.

**Table 4:** Relationship between malaria treatment-seeking time and socio-demographic characteristics in guardians of children with malaria

Variables	Treatment-seeking time				X^2^	*p-value*
	≤ 24 h N_0_ (%)	25–48 h N_0_ (%)	49–72 h N_0_ (%)	≥ 72 h N_0_ (%)		
Gender of guardians						
Male	52 (26.4)	50 (35.7)	7 (31.8)	11 (35.5)	3.711	0.294
Female	145 (73.6)	90 (64.3)	15 (68.2)	20 (64.5)		
Total	197 (100)	140 (100)	22 (100)	31 (100)		
Age of guardians (years)						
20–35	152 (77.2)	89 (63.6)	11 (50.0)	10 (32.3)	35.597	0.000
36–51	41 (20.8)	45 (32.1)	9 (40.9)	15 (48.4)		
52–67	4 (2.0)	6 (4.3)	2 (9.1)	5 (16.1)		
≥ 68	0 (0.0)	0 (0.0)	0 (0.0)	1 (3.2)		
Total	157 (100)	140 (100)	22 (100)	31 (100)		
Marital status						
Monogamy	105 (53.3)	94 (67.1)	9 (40.9)	18 (58.1)	42.213	0.000
Polygamy	3 (1.5)	4 (2.9)	3 (13.6)	3 (9.7)		
Single	29 (14.7)	8 (5.7)	2 (9.1)	0 (0.0)		
Widowed	6 (3.1)	6 (4.3)	1 (4.5)	4 (12.9)		
Divorced/separated	15 (7.6)	1 (0.7)	2 (9.1)	2 (6.5)		
Co-habiting	39 (19.8)	27 (19.3)	5 (22.7)	4 (12.9)		
Total	197 (100)	140 (100)	22 (100)	31 (100)		
Level of education						
Tertiary	117 (59.4)	37 (26.4)	4 (18.2)	3 (9.7)	93.733	0.000
Secondary/high school	73 (37.1)	86 (61.4)	7 (31.8)	16 (51.6)		
Primary	7 (3.5)	17 (12.2)	10 (45.5)	11 (35.5)		
None	0 (0.0)	0 (0.0)	1 (4.5)	1 (3.2)		
Total	197 (100)	140 (100)	22 (100)	31 (100)		

Additionally, treatment-seeking time of guardians for their sick children was found to increase with their household size. A positive and moderate strength correlation (Spearman rho = 0.494; *p* = 0.000) was noted when these two variables were compared.

#### Socio-economic characteristics and treatment-seeking time

Monthly household income and the occupation of guardians were found to be significantly associated with treatment-seeking for malaria. It is noted that the majority of guardians with treatment-seeking time within 24 h had household monthly incomes of at least 101,000 FCFA (Table 5). Private sector employees represented the highest proportion (35.0%) of guardians seeking treatment within 24 h of noticing their child’s symptoms of illness. Government employees made up a smaller 20.3% of this group. Self-employed guardians represented the largest proportion in those seeking care within 25–48 h (48.6%) and within 49–72 h (45.5%).

**Table 5:** Socio-economic characteristics and treatment-seeking time for malaria

Variables	Treatment-seeking time				X^2^	*p-value*
	≤ 24 h N_0_ (%)	25–48 h N_0_ (%)	49–72 h N_0_ (%)	≥ 72 h N_0_ (%)		
Monthly household income (FCFA)						
Low (0–36,000)	1 (0.5)	0 (0.0)	0 (0.0)	0 (0.0)	45.168	0.000
Middle (0–100,000)	46 (23.4)	66 (47.1)	18 (81.8)	16 (51.6)		
High (≥ 101,000)	150 (76.1)	74 (52.9)	4 (18.2)	15 (48.4)		
Total	197 (100)	140 (100)	22 (100)	31 (100)		
Occupation of guardians						
Government employed	40 (20.3)	17 (12.1)	1 (4.5)	3 (9.7)	64.624	0.000
Housewife	13 (6.6)	18 (12.9)	3 (13.6)	3 (9.7)		
Private sector employed	69 (35.0)	33 (23.6)	7 (31.8)	3 (9.7)		
Self-employed	53 (26.9)	68 (48.6)	10 (45.5)	18 (58.1)		
Student	20 (10.2)	1 (0.7)	0 (0.0)	0 (0.0)		
Unemployed	2 (1.0)	3 (2.1)	1 (4.5)	4 (12.9)		
Total	197 (100)	140 (100)	22 (100)	31 (100)		

Furthermore, a positive, moderate strength correlation (Spearman rho = 0.458; *p* = 0.000) was found between the number of children a guardian has under his/her care and their treatment-seeking time for malaria.

### Bivariate analysis of factors favouring delay by guardians to seek hospital treatment of malaria for their children

#### Socio-demographic characteristics that affect delay in seeking treatment

Delay by guardians in seeking malaria treatment for their children was not found to be statistically related to age group or gender. However, compared to guardians having only primary or no education, those with tertiary education were 0.096 times less likely to delay in seeking treatment for their children with malaria (OR 0.096; 95% CI 0.035–0.268). Guardians with a household size of ≤ 5 persons were found to be less likely to delay seeking care for their children (OR 0.117; 95% CI 0.016–0.851) (Table 6).

**Table 6:** Socio-demographic characteristics and treatment-seeking time for malaria

Variables	Overall No. (%)	Treatment-seeking time		OR	95% CI	*p-value*
		Early No. (%)	Delayed No. (%)			
Age groups (years)						
20–35	262 (67.2)	152 (77.2)	110 (57.0)	0.470	0.113–1.963	0.301
36–51	110 (28.2)	41 (20.8)	69 (35.8)	0.635	0.158–2.548	0.522
≥ 52	18 (4.7)	4 (2.0)	14 (7.2)	(Reference)		
Total	390 (100)	197 (50.5)	193 (49.5)			
Gender of guardians						
Female	270 (69.2)	145 (73.6)	125 (64.8)	(Reference)		
Male	120 (30.8)	52 (26.4)	68 (35.2)	1.281	0.710–2. 311	0.412
Total	390 (100)	197 (50.5)	193 (49.5)			
Marital status						
Monogamy	226 (57.9)	105 (53.3)	121 (62.7)	0.962	0.502–1.845	0.907
Polygamy	13 (3.3)	3 (1.5)	10 (5.2)	0.268	0.033–2.179	0.218
Single	39 (10.0)	29 (14.7)	10 (5.2)	0.640	0.254–1.611	0.343
Divorced/separated	20 (5.1)	15 (7.6)	5 (2.6)	0.449	0.115–1.753	0.250
Widowed	17 (4.4)	6 (3.0)	11 (5.7)	1.881	0.485–7.286	0.361
Co-habiting	75 (19.2)	39 (19.8)	36 (18.7)	(Reference)		
TOTAL	390 (100)	197 (50.5)	193 (49.5)			
Level of education						
Tertiary	161 (41.3)	117 (59.4)	44 (22.8)	0.096	0.035–0.268	0.000
Secondary/high school	182 (46.7)	73 (37.1)	109 (56.5)	0.400	0.149–1.078	0.070
Primary/none	47 (12)	7 (3.6)	40 (20.7)	(Reference)		
Total	390 (100)	197 (50.5)	193 (49.5)			
Household size						
≤ 5	266 (68.2)	174 (88.3)	92 (47.7)	0.117	0.016–0.851	0.034
6–9	106 (27.2)	21 (10.7)	85 (44.0)	0.680	0.096–4.844	0.700
≥ 10	18 (4.6)	2 (1.0)	16 (8.3)	(Reference)		
Total	390 (100)	197 (50.5)	193 (49.5)			

*OR* odds ratio

#### Socio-economic characteristics that affect delay in seeking treatment

Compared to unemployed guardians, students were statistically less likely to delay in seeking malaria treatment for their sick children. It was noted that, using high level of income as a reference, guardians of the low/middle income category were 3.6 times more likely to delay seeking hospital-based treatment for malaria (OR 3.641; 95% CI 2.228–5.950). Guardians with at most five children under their care were less likely to seek treatment late (OR 0.097; 95% CI 0.021–0.441) (Table 7).

**Table 7:** Socio-economic characteristics and treatment-seeking time for malaria

Variables	Overall No. (%)	Treatment-seeking time		OR	95% CI	*p-value*
		Early No. (%)	Delayed No. (%)			
Occupation						
Government employed	61 (15.6)	40 (20.3)	21 (10.9)	0.363	0.064–2.052	0.251
Housewife	37 (9.5)	13 (6.6)	24 (12.4)	0.947	0.162–5.552	0.952
Private employee	112 (28.7)	69 (35.0)	43 (22.3)	0.312	0.059–1.647	0.170
Self-employed	149 (38.2)	53 (26.9)	96 (49.7)	0.762	0.146–3.982	0.748
Student	21 (5.4)	20 (10.2)	1 (0.5)	0.022	0.002–0.287	0.004
Unemployed	10 (2.6)	2 (1.0)	8 (4.1)	(Reference)		
Total	390 (100)	197 (50.5)	193 (49.5)			
Monthly income (FCFA)						
Low/middle (0–100,000)	147 (37.7)	47 (23.9)	100 (51.8)	3.641	2.228–5.950	0.000
High (≥ 101,000)	243 (62.3)	150 (76.1)	93 (48.2)	(Reference)		
Total	390 (100)	197 (50.5)	193 (49.5)			
Children under care						
≤ 5	371 (95.1)	195 (99.0)	176 (91.2)	0.097	0.021–0.441	0.003
≥ 6	19 (4.9)	2 (1.0)	17 (8.8)	(Reference)		
Total	390 (100)	197 (50.5)	193 (49.5)			

*OR* Odds ratio

### Multivariate analysis of the factors affecting delay in seeking hospital treatment of malaria for their children

A multivariate logistic analysis was conducted in order to develop a model that could predict the chances of delay in seeking treatment for malaria, in the context of a malaria user fee exemption policy. This analysis made use of all variables with *p-values* less than 0.2 from the bivariate analysis. The variables were: the level of education of guardians, their household size, occupation, estimated monthly household income, number of children under care, and their level of knowledge on the user fee exemption policy.

Table 8 presents the variables considered in the multivariate analysis. The occupation of a guardian was found to be significantly associated with treatment-seeking time for malaria. Compared to being unemployed, students were 0.042 times (AOR 0.042; *p* = 0.020) less likely to delay in seeking hospital treatment for their children with malaria. Additionally, guardians who had attained tertiary education were found to be 0.315 times (AOR 0.315; *p* = 0.036) less likely to delay in seeking hospital treatment for their children with malaria. Guardians with an estimated household income level of low/middle were 3.794 times more likely to delay in seeking treatment compared to those with income estimated as high.

**Table 8:** Determinants of guardians’ delay in seeking malaria treatment for their children

Variables	Overall No. (%)	Treatment-seeking time		AOR	95% CI	*p-value*
		Early No. (%)	Delayed No. (%)			
Occupation						
Government employed	61 (15.6)	40 (20.3)	21 (10.9)	0.392	0.059–2.621	0.334
Housewife	37 (9.5)	13 (6.6)	24 (12.4)	0.629	0.94–4.221	0.633
Private employed	112 (28.7)	69 (35.0)	43 (22.3)	0.418	0.070–2.449	0.339
Self-employed	149 (38.2)	53 (26.9)	96 (49.7)	0.600	0.104–3.451	0.567
Student	21 (5.4)	20 (10.2)	1 (0.5)	0.042	0.003–0.607	0.020
Unemployed	10 (2.6)	2 (1.0)	8 (4.1)	(Reference)		
Monthly income (F CFA)						
Low/middle	147 (37.7)	47 (23.9)	100 (51.8)	3.794	2.125–6.774	0.000
High	243 (62.3)	150 (76.1)	93 (48.2)	(Reference)		
Level of education						
Tertiary	182 (46.7)	73 (37.1)	109 (56.5)	0.315	0.107–0.927	0.036
Secondary/high school	47 (12)	7 (3.6)	40 (20.7)	0.637	0.239–1.697	0.367
Primary/none	161 (41.3)	117 (59.4)	44 (22.8)	(Reference)		
Number of children under care						
≤ 5	371 (95.1)	195 (99.0)	176 (91.2)	0.510	0.072–3.611	0.500
≥ 6	19 (4.9)	2 (1.0)	17 (8.8)	(Reference)		
Household size						
≤ 5	266 (68.2)	174 (88.3)	92 (47.7)	0.141	0.019–1.055	0.056
6–9	106 (27.2)	21 (10.7)	85 (44.0)	1.060	0.146–7.709	0.954
≥ 10	18 (4.6)	2 (1.0)	16 (8.3)	(Reference)		

## Discussion

This study sought to determine the factors favouring delay by guardians seeking hospital-based treatment for their children with malaria, despite the existence of a user fee exemption. It provides baseline information that could be used to strengthen policies geared towards access to health facilities.

### Health-seeking behaviour of guardians of children with malaria

A majority (62.1%) of guardians in the study administered some form of treatment to their children before seeking hospital-based care. This is similar to findings from other studies carried out in Nigeria, Laos, Ethiopia, and Cameroon, where guardians were noted to use homemade remedies, drugs from roadside vendors and/or herbs before seeking hospital-based treatment when their child did not get better [24–27]. Even though all age groups in the study made considerable use of non-hospital-based remedies, the 20–35 years age group was least likely to try pre-hospital treatments. This could be explained by the fact that this age group is made up of young and new parents who are more cautious about their children’s health, and prefer to directly seek care at an established health facility.

Similar to a study in the general population of Cameroon depicting high levels of self-medication in adults [28], this study also found that non-hospital-based care of sick children was mostly done by self-medication. However, in other studies, the most prominent pre-hospital treatment-seeking behaviour was the use of potions from traditional medicine practitioners [26, 29]. The tendency to first seek care from traditional medicine practitioners in these latter studies could be due to the fact that the study areas were rural and guardians were less likely to obtain over-the-counter drugs from pharmacies or roadside vendors. Indeed, the second most common pre-hospital treatment-seeking behaviour in the study was consulting a pharmacy, while seeking care from a herbalist was mentioned by 8.3% of the guardians who sought out-of-hospital remedies.

### Reasons for delay in seeking hospital-based treatment for children with malaria

About half (50.5%) of guardians in the study brought their children to hospital-based care for malaria within 24 h of noticing symptoms of illness. This is in contrast to other studies in Southern Ghana and Eastern Nigeria that reported 11% and 22%, respectively, of guardians seeking hospital treatment within 24 h [10, 30]. This difference could be due to the fact that these latter studies were carried out in rural localities where the belief in traditional remedies is stronger than in the more urban study area. This point is supported by an Ethiopian study which noted that guardians from urban communities were more likely to seek hospital-based care than their rural peers [26]. The current study area has a considerable number of health facilities at proximity to the population. This could have contributed to more of our study participants seeking treatment earlier in hospitals.

Seeking hospital treatment after 24 h was noted in 49.5% of the participants. The main reasons mentioned for this delay were financial constraints, followed by the need to observe the sick child for a while, hoping he/she got better. This latter reason was mostly given by those who administered some non-hospital-based treatment to their children. A similar study in Nigeria also mentioned watchful waiting as a major reason why guardians delayed seeking hospital treatment for their children with malaria [10]. In the general population of Cameroon, financial barriers have been pointed out as the most significant challenge in seeking appropriate treatment from health facilities [31]. Interestingly, none of the guardians in this study mentioned distance to health facilities as a hurdle to seeking treatment at hospitals. This finding contrasts with that of other studies that noted distance and accessibility to health facilities were a determinant of treatment-seeking time for malaria in children [11, 30]. The study area, being an urban setting, is served with several health facilities distributed all over the health district. Access to hospitals is mostly easy. In 2% of participants, other reasons for delay such as insecurity prevented free movements to seek care, and the guardian being sick were mentioned.

### Social factors on the treatment-seeking time of guardians of children with malaria

Factors affecting malaria treatment-seeking time were classified as early (≤ 24 h) or delayed (≥ 25 h), in the context of a user fee exemption. After a bivariate analysis was run, predictors with *p-values* less than 0.2 were used to create a multivariate logistic regression model, adjusting for potential confounders. It was noted that the educational level of guardians was a significant predictor of the time taken to seek care at a health facility. Guardians with a tertiary level of education were less likely to delay in seeking treatment for their children. This conforms to similar studies in Nigeria [10, 32]. This could be explained by the fact that educated guardians are more likely to quickly recognize the signs and symptoms of malaria in their children and make the decision to seek adequate health care in a health facility.

The monthly household income proved to be a predictor of malaria treatment-seeking time by guardians. Guardians with an estimated monthly income above 100,000 F CFA were less like to delay seeking treatment for their children. Chukwuocha et al. [10] in Nigeria had similar findings. This was particularly valid in this Nigerian study, which did not look at a population with a user fee exemption policy, as guardians had to make out-of-pocket payments in order for their children to benefit from malaria treatment. However, in the current study population, the existence of a user fee exemption was logically supposed to help guardians seek treatment even when they had a low monthly income. This inconsistency could be explained by the fact that there seem to exist other auxiliary costs to malaria treatment which are not covered by the user fee exemption policy. A 2015 study in the Adamawa Region of Cameroon noted the existence of informal costs for malaria treatment at various health facilities, even though all services were supposed to be free [17]. This could act as a deterring factor, preventing guardians with low income from seeking hospital-based malaria treatment. The study found the occupation of guardians to be a determinant of their treatment-seeking time. When compared to their unemployed counterparts, students were significantly least likely to delay in seeking malaria treatment for their children at health facilities. This finding conforms to a similar study from Nigeria and Ethiopia [10, 26].

This study showed that most (73.6%) guardians who sought early treatment for their children were female. Though gender was not a statistically significant determinant of early treatment-seeking, this trend could be that most caregivers are female and are, therefore, more predisposed to accompany their children to the hospital.

## Limitations

The time taken by guardians to seek hospital treatment was an estimate and could have been affected by a recall bias. The study did not take into consideration the ability of guardians to recognize malaria and its severity in their children. This could have played a role in affecting treatment-seeking behaviour and time to seek hospital treatment. However, to the best of our knowledge, this study is the first in Cameroon to determine the factors affecting the time taken by guardians to seek malaria treatment in health facilities for children under 5 years, in the context of a user fee exemption.

## Conclusions

Despite the current policy on free malaria treatments for children under 5 years old in health facilities, most guardians of children with malaria remain reluctant to seek adequate treatment in health facilities. This delay is found to be determined by social and economic factors inherent in guardians. The current user fee exemption policy is therefore not sufficient to stimulate considerable malaria treatment-seeking in health facilities. There is a need to ameliorate the social and economic welfare of the population in order to decrease delay in seeking malaria treatment from health facilities.

## Data Availability

All data generated or analysed during this study are included in this published article and its information files.

## Questionnaire

**Table:**

1	Date of interview		dd/mm/yyyy	___/___/_____/
**Part 1: Identification and malaria suspicion**				
2	Note participant’s code		│___│__│___│___│	
3	Participant’s phone number		│_______________│	
4	What is the age of your child (years)?		│___│__│	
5	What is the gender of your child?		Male = 1 Female = 2	│___│
6	Did you suspect that your child had malaria before coming to the hospital?		Yes = 1 No = 2	│___│
**Part 2: Socio-demographic characteristics**				
7	How old are you? (years)		│___│__│	
8	What is your gender?		Male = 1 Female = 2	│___│
9	What is your marital status?		Married (monogamy) = 1 Married (polygamy) = 2 Single = 3 Divorced/separated = 4 Widow/widower 5 Co-habiting = 6	│___│
10	What is your highest level of education?		Never attended school = 1 Primary = 2 Secondary/ high school = 3 Higher education = 4	│___│
11	Where do you live?		│_______________│	
12	How many persons live in your house?		│___│___│	
13	What is your religion?		None = 1 Christian = 2 Muslim = 3 Other = 4	│___│
				If 4, state it:
**Part 3: Socio-economic characteristics**				
14	How many children are there under your care?		│___│___│___│	
15	What do you do for a living?		Unemployed = 1 Housewife = 2 Student = 3 Self-employed = 4 Employed in private sector = 5 Employed in government = 6	│___│
16	What is the monthly income for your household? (FCFA)		Low (0–36,000) = 1 Middle (37,000–100,000) = 2 High (Above 101,000) = 3	│___│
**Part 4: Treatment-seeking behaviour and time**				
17	Did you seek other sources of treatment before bring your child to the hospital?		No = 1 Yes = 2	│___│
18	If yes in 17 above, where did you seek for treatment?		Self-medication = 1 Roadside vendor = 2 Pharmacy = 3 Herbalist = 4 Other hospital = 5 Family/friend = 6 Other = 7	│___│ │___│ │___│ │___│ │___│ │___│ │___│
				If 7, explain:
19	19a	How much time elapsed, from when you noticed your child’s first symptoms till when you brought him/her to the hospital?	│___│___│___│hours	< / = 24 h (Early) > 24 h (Delayed)
	19b	If 19a is above 24 h, what is the reason (s) for the delay?	Watch the child for a while = 1 Financial constraints = 2 Health facility is far = 3 Use of alternative care = 4 Other = 5	│___│ │___│ │___│ │___│
